# Playing 20 Questions with the Mind: Collaborative Problem Solving by Humans Using a Brain-to-Brain Interface

**DOI:** 10.1371/journal.pone.0137303

**Published:** 2015-09-23

**Authors:** Andrea Stocco, Chantel S. Prat, Darby M. Losey, Jeneva A. Cronin, Joseph Wu, Justin A. Abernethy, Rajesh P. N. Rao

**Affiliations:** 1 Department of Psychology, University of Washington, Seattle, Washington, United States of America; 2 Institute for Learning & Brain Sciences, University of Washington, Seattle, Washington, United States of America; 3 Department of Computer Science & Engineering, University of Washington, Seattle, Washington, United States of America; 4 Department of Bioengineering, University of Washington, Seattle, Washington, United States of America; UCLA, UNITED STATES

## Abstract

We present, to our knowledge, the first demonstration that a non-invasive brain-to-brain interface (BBI) can be used to allow one human to guess what is on the mind of another human through an interactive question-and-answering paradigm similar to the “20 Questions” game. As in previous non-invasive BBI studies in humans, our interface uses electroencephalography (EEG) to detect specific patterns of brain activity from one participant (the “respondent”), and transcranial magnetic stimulation (TMS) to deliver functionally-relevant information to the brain of a second participant (the “inquirer”). Our results extend previous BBI research by (1) using stimulation of the visual cortex to convey visual stimuli that are privately experienced and consciously perceived by the inquirer; (2) exploiting real-time rather than off-line communication of information from one brain to another; and (3) employing an interactive task, in which the inquirer and respondent must exchange information bi-directionally to collaboratively solve the task. The results demonstrate that using the BBI, ten participants (five inquirer-respondent pairs) can successfully identify a “mystery item” using a true/false question-answering protocol similar to the “20 Questions” game, with high levels of accuracy that are significantly greater than a control condition in which participants were connected through a sham BBI.

## Introduction

Direct brain-to-brain interfaces (BBIs) are technologies that combine neuroimaging and neurostimulation methods to exchange information between brains directly in neural code. In BBIs, specific content is extracted from the neural signals of a “sender” brain, digitized, and re-encoded in the form of induced neural activity in a “receiver” brain. BBIs have been recently demonstrated in both animal models [1–3] and humans [4–6]. While BBIs in humans offer advantages in terms of the range of tasks that can be accomplished and the complexity of information that can be transferred [7], constraints on the invasiveness of brain stimulation technologies in humans limit the possible BBI paradigms. As a result, all of the existing human BBIs have adopted two standard, non-invasive, and relatively safe technologies; electroencephalography (EEG [8]) to record brain activity, and transcranial magnetic stimulation (TMS [9]) to modulate neural activity. Using these two technologies, Rao and colleagues [5] were able to detect the intention of moving the right hand in a sender, and induce the intended movement in the right hand of a receiver, thus allowing the pair to jointly cooperate to solve a visuo-motor task. Grau and colleagues [6], on the other hand, were able to transfer information between pairs of participants in the form of neurally modulated visual percepts, which could be decoded as words by the receiver through a pre-arranged code.

While both experiments served as important proofs of concept, they were also subject to a number of limitations. For example, in Rao and colleagues’ study [5], the participant at the receiver end of the BBI received a stimulus to the motor cortex, which produced the intended motor action but was not consciously processed by the participant. In other words, the receiver did not experience the desire to move his or her hand. Therefore, while the BBI allowed two participants to collaborate to achieve a common goal, it did not take full advantage of the receiver’s capacity for processing information. In Grau and colleagues’ experiment [6], the information transmitted to the receiver through the stimulation of visual processing areas resulted in the conscious perception of “phosphenes,” which are temporary visual percepts in the form of lines or spots. The conscious experience of phosphenes was interpreted as a binary signal and used to encode simple words with a code akin to the Morse code. This approach takes full advantage of the human capacity to consciously process information, but because their BBI was not intended to achieve a common goal between the two participants, it lacked the collaborative nature of Rao and colleagues’ task [5]. Also, differently than Rao’s study, Grau and colleagues adopted an off-line delivery mode, where the same signals from a single sender participant were recorded and later broadcast to different receivers. Finally, none of the previous studies had the two participants engage in collaborative and interactive problem solving, in which the receiver can transmit information back to the sender. Such a “closed-loop” interaction could represent a significant advance for the potential applications of human BBI technologies.

We report results from a new experiment that overcomes the limitations of these previous studies by increasing both the complexity of the tasks performed with a non-invasive BBI in humans, and the quantity of information transferred between two brains. In our experiment, five pairs of participants played a shortened version of the popular “20 Questions” parlor game, in which a participant can ask up to 20 yes/no questions in order to figure out what object a second participant is thinking of. We chose this paradigm because it presents several important features that test the utility of a BBI, including the fact that it is collaborative, potentially open-ended, operates in real-time, and requires conscious processing of incoming information.

**Fig 1:**
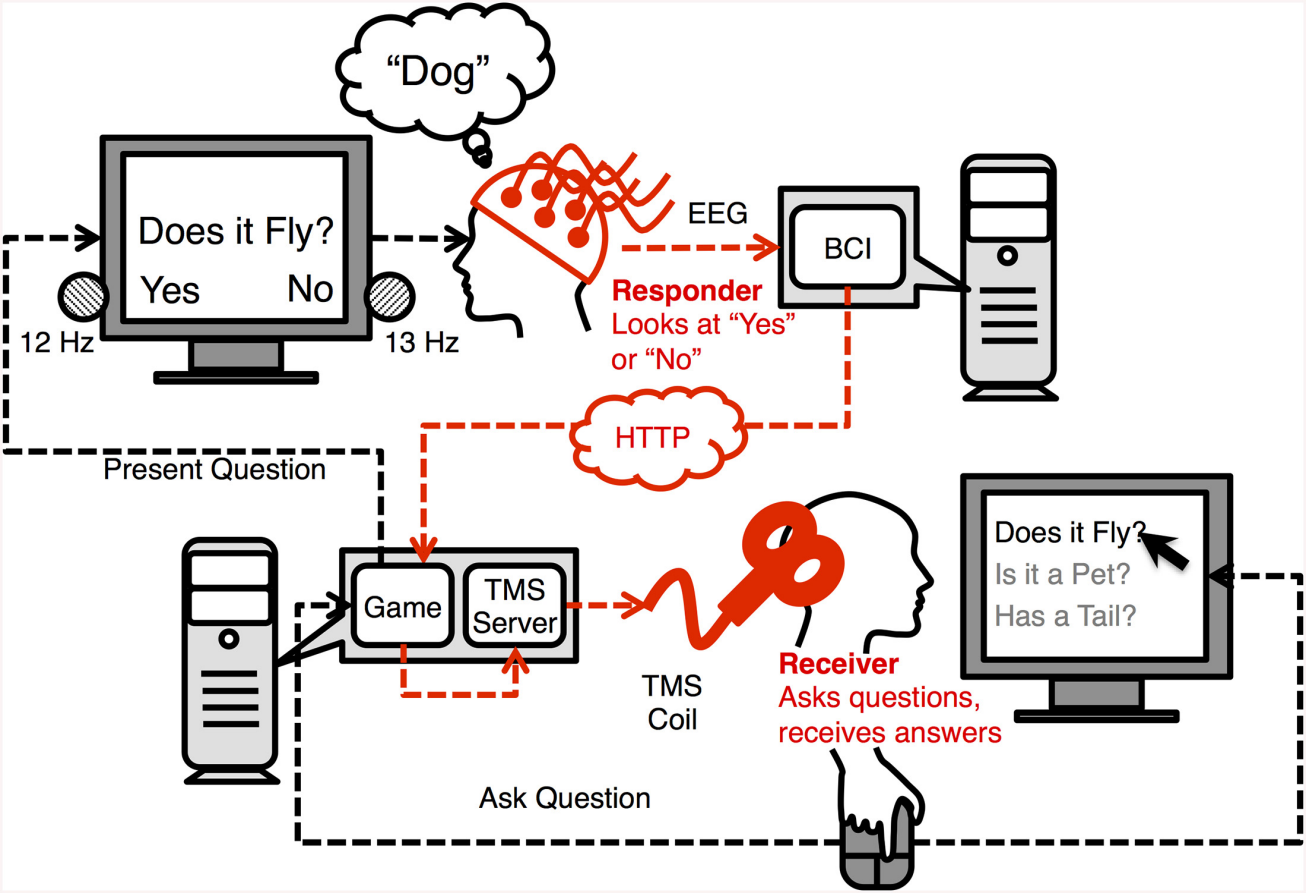
Architecture of the BBI and “20 Questions” Experiment. In the experiment, two participants (an “inquirer” and a “respondent”) played a question-answering game similar to “20 Questions.” The respondent is given an object (e.g., “dog”) that is unknown to the inquirer and that the inquirer has to guess. The inquirer asks a question about the object by selecting a question (using a mouse) from questions displayed on a screen. The question is then presented visually to the respondent through a web interface. The respondent answers “Yes” or “No” directly through their brain signals by paying attention to one of two flashing LEDs (“Yes” = 13 Hz; “No” = 12 Hz). The BBI uses EEG to decode the respondent’s answer, and a TMS apparatus to convey the answer to the inquirer by generating a visual percept through stimulation for “Yes” and the absence of a percept for “No.” In the figure, the BBI system is highlighted in red.

To better reflect their roles in the collaborative question-answering task, we refer to the BBI sender as “Respondent”, and the BBI receiver as “Inquirer.” As illustrated in Fig 1, the inquirer and the respondent (physically located about 1 mile apart) interact using a web interface in conjunction with the brain-to-brain interface. The respondent first thinks of an object belonging to a specific category (for example, an animal, e.g., “dog”); the inquirer then uses the web interface to select a question for the respondent (using a computer mouse) from a list shown on the computer screen. A non-invasive BBI (shown in red in Fig 1) decodes the answer directly from the respondent’s brain signals and conveys the answer to the inquirer through stimulation.

The BBI was designed to detect visual information from the respondent’s EEG activity (specifically, whether the respondent was attending to the “Yes”-labelled flashing LED or the “No”-labeled flashing LED), and elicit a corresponding visual percept in the inquirer through transcranial magnetic stimulation of the visual areas in the occipital cortex. A number of precautions (see below) were taken to ensure that the inquirer did not receive any information about the answer from any source other than the BBI itself. To ensure the validity of these precautions, each experimental session consisted of 10 experimental games and 10 “control” games; during the control games, the BBI sounded and felt similar to the experimental trials but it did not stimulate the occipital cortex. The order of experimental/control games was randomized prior to the beginning of the experiment. Participants were told in advance about the presence of two conditions, but were not told which condition each game belonged to.

## Materials and Methods

### Human Subjects and Ethics Statement

Ten healthy participants (aged 19–39 yrs, seven female; see Table 1), took part in a controlled laboratory experiment. To achieve the planned number of 10 participants, 12 subjects were originally recruited, but two could not complete the experiment because of either problems with the equipment (in the case of 1 respondent, female, aged 29) or because the phosphene threshold could not be determined (in the case of 1 inquirer, male, aged 28).

**Table 1:** Participants demographics. Age, gender, and role of all the participants who completed the experiment.

Pair	Role	Gender	Age
**1**	Respondent	*F*	26
	Inquirer	*M*	38
**2**	Respondent	*F*	19
	Inquirer	*F*	39
**3**	Respondent	*F*	32
	Inquirer	*M*	30
**4**	Respondent	*F*	26
	Inquirer	*F*	31
**5**	Respondent	*M*	24
	Inquirer	*F*	28

All the participants who completed the experiment were included in the analysis. All participants were recruited through word-of-mouth advertisement from the student, staff, and faculty population at the University of Washington. The ten participants were divided into five pairs, with one participant playing the role of the respondent and one playing the role of the inquirer. With the exception of the two participants in Pair #2, none of the pairs of participants knew each other. Because the TMS procedure is inherently riskier than the EEG procedure, participants were allowed to decide which role they wanted to play. To maintain their decision free of any external influence, all participants received monetary compensation that was independent of their role and proportional to the total amount of time devoted to the study. Both the experimental and the recruitment procedures were reviewed and approved by the Institutional Review Board of the University of Washington (IRB Application #46065). All participants were fully informed about the experimental procedure and its potential risks and benefits and gave written consent prior to the beginning of the experiment. The individuals in this manuscript have also given written informed consent to publish their case details.

### Experimental Task

In our simplified version of the 20 Questions game, questions and objects were pre-defined so that each game could be solved in exactly three consecutive question-and-answer cycles. At the beginning of each game, the inquirer selected one list of objects from a set of 20 different lists. Each list contained eight object names and an associated set of three yes/no questions. All of the respondents were given the opportunity to ask the experimenter any questions if they were not familiar with the selected object, but ultimately no respondent did so. The questions were designed so that each object was associated with one specific combination of “Yes” and “No” answers to each question (see Table 2 for an example).

**Table 2:** The “Animals #1” Set. An example of the sets of objects (first column) and associated questions used during the experiment.

Target	Can it fly?	Is it a mammal?	Is it a pet?
**Shark**	No	No	No
**Turtle**	No	No	Yes
**Bear**	No	Yes	No
**Dog**	No	Yes	Yes
**Vulture**	Yes	No	No
**Parakeet**	Yes	No	Yes
**Bat**	Yes	Yes	No
**Sugar glider**	Yes	Yes	Yes

After a list was chosen, one object from the list was randomly selected by the software and presented as the object of choice to the respondent, but kept hidden from the inquirer. The inquirer was instead shown a list of possible objects on the right side of the computer screen and an associated list of questions on the left side. A question-and-answer cycle was initiated when the inquirer selected one of the questions with a mouse. The inquirer’s screen was then changed to a fixation cross, while at the same time, the question appeared on the respondent’s computer screen.

The respondent answered the question about the object by shifting attention to either a “Yes” or a “No”-labeled flashing LED adjacent to the computer screen (see Fig 1). The respondent was allotted 2 seconds to read the question, 1 second to choose an answer and begin looking at the corresponding LED light, and 20 seconds to complete the trial, during which the respondent’s EEG brain activity was recorded and analyzed in real-time to determine which of the two LEDs he or she was attending to. During this interval, the inquirer waited for a corresponding neural signal to be delivered via stimulation through the BBI. The signal took the form of a visual percept, which was elicited by stimulating the inquirer’s visual cortex, and whose appearance signified a “Yes” answer from the respondent and whose absence indicated a “no” answer. At the end of the waiting period, the inquirer was asked to indicate with a mouse press whether he or she had seen the elicited visual percept; his or her answer was then taken as the respondent’s answer to the question about the object. The inquirer was then presented with an updated list of possible objects and questions, so that all previous questions and all the animals excluded by the most recent answer were grayed out. Such a question-and-answer cycle was repeated three times, at the end of which only one object was left, and recorded as the inquirer’s final guess. At this point, a visual message informed the respondent (but *not* the inquirer; see below) of the correctness or incorrectness of the guess. A new game began immediately thereafter.

As illustrated in the example in Table 2, the answer to each question evenly divides the set into four objects with a positive answer and four with a negative answer, and their properties are arranged so that each object is associated with a unique combination of the three answers. Thus, within each set, the answer to each question conveys exactly one bit of information, and each object can be identified with three unique bits. This permits us to easily quantify the amount of information that was transferred within each pair.

During the experiment, the respondent and the inquirer were located approximately 1 mile apart, in different buildings on the University of Washington campus: the respondent was located in the department of Computer Science and Engineering, while the inquirer was located in the Institute for Learning and Brain Sciences. Each experimental session lasted approximately 50 minutes, and consisted of 10 experimental games and 10 control games (see below), the order of which was randomized prior to the beginning of the experiment.

### Brain to Brain Interface

As in our previous study [5], the direct brain-to-brain interface was built out of two non-invasive technologies: EEG [8] for recording brain signals from the scalp and TMS [9] for indirectly modulating neural activity through rapidly changing magnetic fields.

The respondent’s answer was identified from real-time EEG recordings using steady-state visually-evoked potentials (SSVEPs), which are stable oscillations of neural activity in the visual cortex that match the frequency of flashing visual stimuli [10], and which yield among the highest information transfer rates in non-invasive brain-computer interfaces [11]. To elicit different SSVEPs, two LED lights were located beside the “Yes” and “No” labels on the computer screen, flashing at characteristic frequencies of 13 Hz and 12 Hz respectively (Fig 1). To provide real-time feedback to the respondent, the characteristic frequency of the “Yes” and “No” LEDs was used to control the position of a 1D cursor, so that power increases in the frequency associated with the “Yes” LED moved the cursor towards the “Yes” label, while power increases in the frequency of the “No” LED moved the cursor towards the “No” label. The respondent’s answer was determined when the cursor reached the edge of the screen corresponding to either the “Yes” or “No” position, or alternatively, if the maximum trial duration of 20 seconds was exceeded, the respondent’s answer was taken to be the answer that the cursor was closest to.

The respondent’s answer was subsequently translated into a single TMS pulse delivered to the occipital cortex of the inquirer. Neurostimulation of the primary and secondary visual areas in the occipital cortex elicits visual percepts known as “phosphenes” [12,13], which are subject-specific but consistently perceived as flashes of light, blobs, or lines in specific locations of the visual field. The intensity of the TMS pulse was manipulated so that it could be clearly interpreted as a binary response. A “Yes” answer from the respondent was translated into an above-threshold pulse, causing the perception of a phosphene; a “No” answer, on the other hand, was translated into a below-threshold pulse, which did not elicit a corresponding phosphene.

Three steps were taken to ensure that all the information from the respondent to the inquirer was traveling only along the BBI and was not contaminated by other sensory cues. First, the subject always received stimulation on the same spot of the scalp, independent of the respondent’s answer; the only difference between “Yes” and “No” answers was that the former was associated with an above-threshold response, and the latter was associated with a below-threshold response. Because different stimulation intensities are also associated with different levels of noise, participants were required to wear earplugs (with a nominal noise cancelation effect of 33 dB). Because noise also travels through the skull bone, the stimulation intensities were slightly varied from trial to trial in increments of 2%. Thus, if a subject’s “high” threshold was 50% and the “low” was 40%, the stimulation intensities were randomly chosen between 50%, 52%, and 54% for high, and 36%, 38%, and 40% for low. This variability further decreased the reliability of sound as an indicator of pulse intensity.

As a third and final precaution, 10 control games were randomly intermixed within 10 experimental games. During experimental games, the non-invasive BBI channel was fully operational. During control games, the BBI was made non-operational by reducing the effect of the TMS coil’s induced magnetic field. In the TMS literature, it is customary to create control conditions by either using a different stimulation site or a specially designed “sham” coil. Neither option, however, is sufficiently similar to the experimental condition to remain undetected by the inquirer. Specifically, participants can easily detect when a functioning coil is placed on a different location, and sham coils are designed not to produce *any* of the peripheral sensory stimulation (such as the coil vibration or peripheral nerve stimulation), associated with the magnetic induction. To overcome these problems, we chose to reduce the efficacy of the magnetic field in the control conditions by attaching a custom-made clip-on plastic module to the TMS coil, whose surface was designed to mimic the shape and texture of the coil. The module effectively separated the TMS coil from the scalp by 20mm. This distance prevented the appearance of phosphenes while largely maintaining the peripheral sensory cues (i.e., noise and scalp sensation) that are associated with a TMS pulse. Note that the electromagnetic pulse was still delivered at the same intensities, so that the two conditions felt and sounded sufficiently similar that the participants were not able to distinguish them. Additionally, the inquirer was not told about the correctness of their final guess, thus preventing the opportunity to identify any other possible cues associated with the two conditions by means of feedback.

Only the experimenter on the TMS side was aware of whether each game was an experimental or control trial. Both participants, as well as the experimenter on the EEG side, were blind to the current game’s condition.

### EEG Procedure for the Respondent

**Fig 2:**
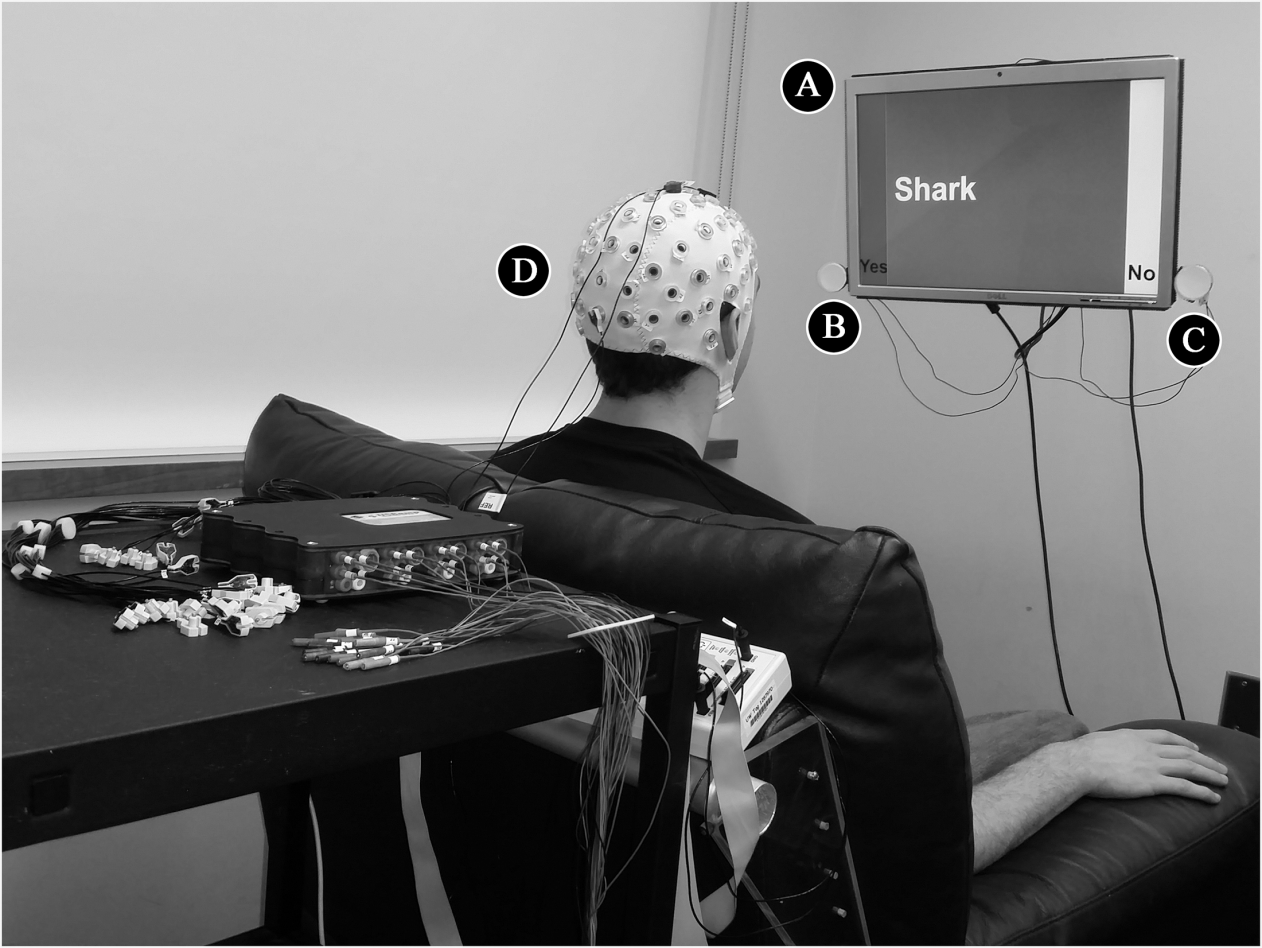
EEG Components of the BBI for the Respondent. During the experiment, the respondent sat in front of a computer screen (A) on a comfortable chair, while EEG signals were recorded from the scalp using an active electrode cap (D). SSVEPs in the EEG signal from the occipital lobe were driven by the frequencies of two flashing LEDs, positioned on the left (“Yes” answer, 13 Hz: B) and the right (“No” answer, 12 Hz: C) side of the screen.

Participants playing the role of the respondent were fitted with an EEG cap and seated in front of a monitor (Fig 2A) with “Yes” and “No” text labels at the lower left and right corners, respectively. Two flashing LED lights were adjacent to the labels; the “Yes” LED (Fig 2B) flashed at 13 Hz, while the “No” LED (Fig 2C) flashed at 12 Hz. During the session, electrical signals were recorded at a frequency of 512 Hz from the respondent’s scalp via a 64-channel Ag/AgCl electrode cap (actiCAP, Brain Products GmBH, Gilching, Germany; Fig 2D) and amplified using gUSBamps (Guger Technologies, Austria).

The decision to use 12Hz and 13 Hz was made based on experiments prior to running the first subject. These frequencies yielded both the highest accuracy and quickest classification. A larger difference between the frequencies would not have necessarily improved accuracy as many previous experiments have demonstrated effective SSVEP-based BCIs with frequency differences less than or equal to 1 Hz [14,15], with some using frequency differences as small as 0.1 Hz [16].

Signals were recorded from an occipital electrode (Oz in the 10–10 placement system), while two frontal electrodes, AFz and FCz (both in the 10–10 system), were used as the ground and reference electrodes, respectively. SSVEP signals were detected using the Fast Fourier Transform with frequencies for spectral power binned from 0 to 17 Hz with 1 Hz bin widths. Respondents did not receive any training before beginning the task. An increase in the spectral power of the 13 Hz bin moved the horizontal cursor towards the “Yes” side, while an increase in the spectral power of the 12 Hz bin moved the cursor towards the “No” side. This SSVEP-controlled visual interface can be thought of as accumulating evidence for “Yes” or “No” answers based on classification of EEG spectral power, with the edges of the screen representing the confidence thresholds for either the “Yes” or “No” answer. If this confidence threshold was not reached within the allotted 20 seconds, the answer corresponding to the closer edge (that is, the answer with higher confidence level) was chosen. In the experiments, across all subjects, 38% of the answers were chosen in this way (i.e., after the allotted time elapsed). Signal processing and data storage were managed through the BCI2000 software package [17].

### TMS Procedure for the Inquirer

Before the experiment, the inquirer underwent a TMS training session, which lasted between 1 and 2 hours. During the training session, the inquirer wore a tight-fitting swim-cap, over which a grid was traced by first marking the inquirer’s inion, and then marking 8 points located on an imaginary 3x3cm grid whose bottom-right corner was the inion. Each marked location was then tested to identify the most reliable stimulation site for eliciting phosphenes. The position of each inquirer’s stimulation site is reported in Table 3. The stimulation values for the high and low intensities were determined by administering 10 pulses at different intensities, from 35% to 70% of the stimulator output, and tabulating the probability that the inquirer reported seeing a phosphene for each level. The above-threshold intensity was determined as the smallest intensity at which the participant reported seeing a phosphene for all 10 stimulations at that intensity. Conversely, the below-threshold intensity was identified as the largest intensity at which the participant never saw a phosphene. The values of these two intensities were determined individually for each inquirer and are reported in Table 3.

**Table 3:** TMS Stimulation Parameters. TMS stimulation intensities and sites for the five inquirers in the experiment. Intensities are expressed as percentage of the stimulator’s maximum output.

Inquirer’s Pair	Above-Threshold Intensity	Below-Threshold Intensity	Stimulation Site (relative to inion)
**1**	58%	41%	2 cm above, 1 cm left
**2**	62%	46%	2 cm above, 1 cm left
**3**	65%	48%	2 cm above, 1 cm left
**4**	67%	52%	2 cm above, 1 cm left
**5**	69%	43%	3 cm above

**Fig 3:**
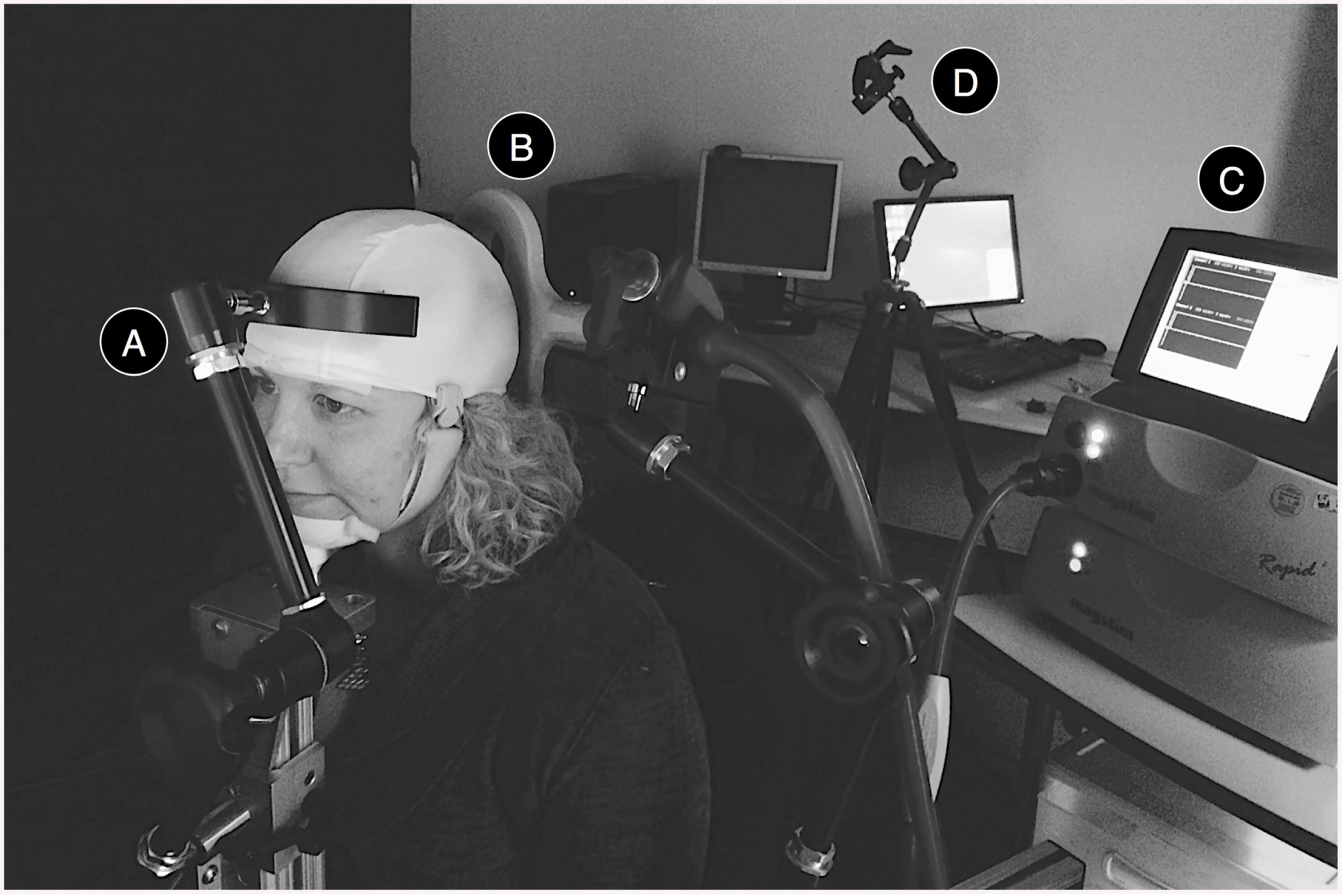
TMS Components of the BBI for the Inquirer. During the experiment, the inquirer sat in front of a computer screen on a BrainSight chair, with his or her head kept in place by a two-pronged head rest (A). A figure-of-8 TMS coil (B), connected to a MagStim Super Rapid^2^ stimulator (C) was positioned over the inquirer’s occipital lobe to deliver visual stimulation in accordance with the respondent’s answer. The head position and the coil position were carefully checked with the aid of a laser pointer (D).

During the experiment, the inquirer sat in a darkened room, wore high-quality noise-cancelling earplugs, and was accommodated on a BrainSight chair (Rogue Research, Montreal, CA), with his or her chin placed over a chin rest and kept in place against a forehead support (Fig 3A). A 70-mm figure-of-8 coil (Double 70mm Alpha coil, Magstim UK) was then placed over his or her occipital lobe (Fig 3B), and connected to a biphasic transcranial magnetic stimulator (Super Rapid^2^, Magstim, UK; Fig 3C). At the beginning of each game, the experimenter checked the absolute head position and the relative coil position by means of a laser beam that was pointed to the inquirer’s target stimulation site (Fig 3D), and made the necessary adjustments. If the next game was a control game, the experimenter also attached the clip-on prop to the TMS coil before its placement over the occipital lobe. To prevent the inquirer from identifying the two conditions based on the pre-game preparations, the coil was repositioned at the beginning of every game. During the repositioning, the experimenter stood behind the participant’s back so as not to be seen, and the timing of the procedure operations was maintained constant across trials.

To facilitate the perception of phosphenes by maintaining a consistent level of ambient light, both the training and the experimental procedure took place in a darkened room. Participants were given 10 minutes to habituate to the light conditions before the beginning of the training session.

## Results and Discussion

The efficacy of the proposed BBI was quantified using several measures. The most straightforward is the number of games in which the inquirer was successfully able to identify the object that the respondent was thinking of. On average, our five pairs of subjects were able to successfully guess the correct object in 72% of the games in the experimental condition using information available from the BBI, in contrast to only 18% successfully guessed during the control conditions. Because the distributions of correct guesses in both the experimental and control conditions were Normal (as assessed by the Shapiro-Wilk Normality test: *W* > 0.91, *p* > 0.48) and the two distributions had equal variances (Levene’s test: *F*(1, 8) = 0.07, *p* = 0.79), the difference between conditions was tested with a paired *T*-test. he difference between the experimental and control conditions was highly significant (paired *t*(4) = 13.50, *p* = 0.0002, Cohen’s *d* = 9.55).

**Fig 4:**
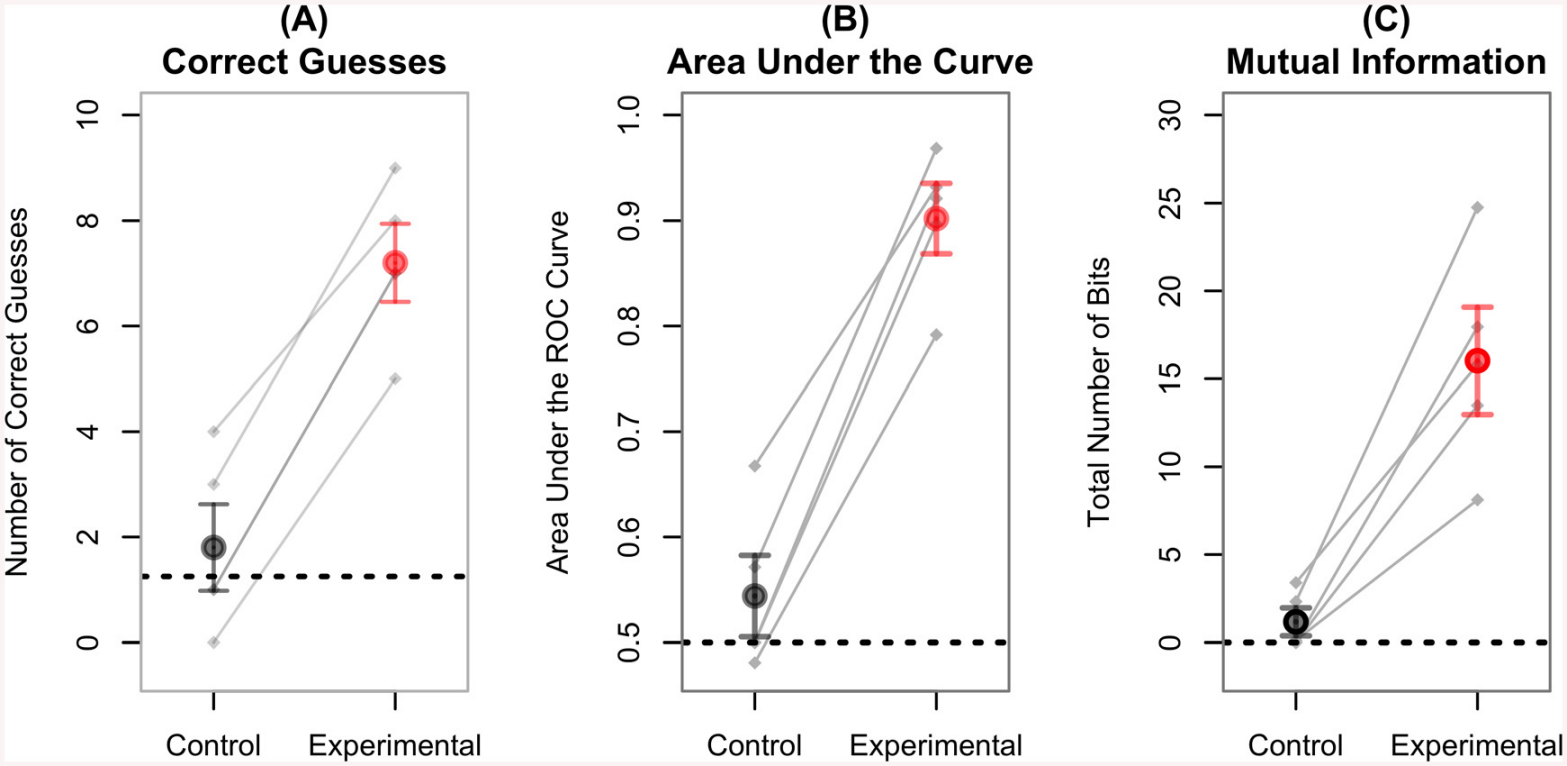
Different Measures of BBI Performance Across Conditions and Subjects. (A) Mean number of objects correctly guessed by the inquirer over 10 experimental (red) and 10 control trials (black) across 5 pairs of subjects; chance performance is 0.125 (dotted line); (B) Mean area under the ROC curves (see Fig 5, below); chance performance is 0.5 (dotted line); (C) Mean number of bits transferred during the experimental and control conditions, using the mutual information criterion; chance performance corresponds to 0 bits (dotted line). In all figures, the grey lines represent the five pairs of participants. Note that in (A), two pairs near the center of the plot had identical performance, giving the appearance of only four lines.

When examining the control condition, it is important to measure chance performance. Chance performance can be estimated in two ways, either by assuming a random inquirer who classifies the respondent’s answers as “Yes” or “No” with equal probability, or assuming an ideal inquirer who always classifies the respondent’s answers as “No” because no cortical input is received in the control condition. Because of the specific ways in which our lists and questions were constructed, both estimates yield the same chance performance of 12.5%. Statistical analysis revealed that performances of all participants were above chance in the experimental condition (single-sample *t*(4) = 8.97, *p* = 0.0008), but not in the control condition (single-sample *t*(4) = 0.78, *p* = 0.50; Fig 4A).

**Fig 5:**
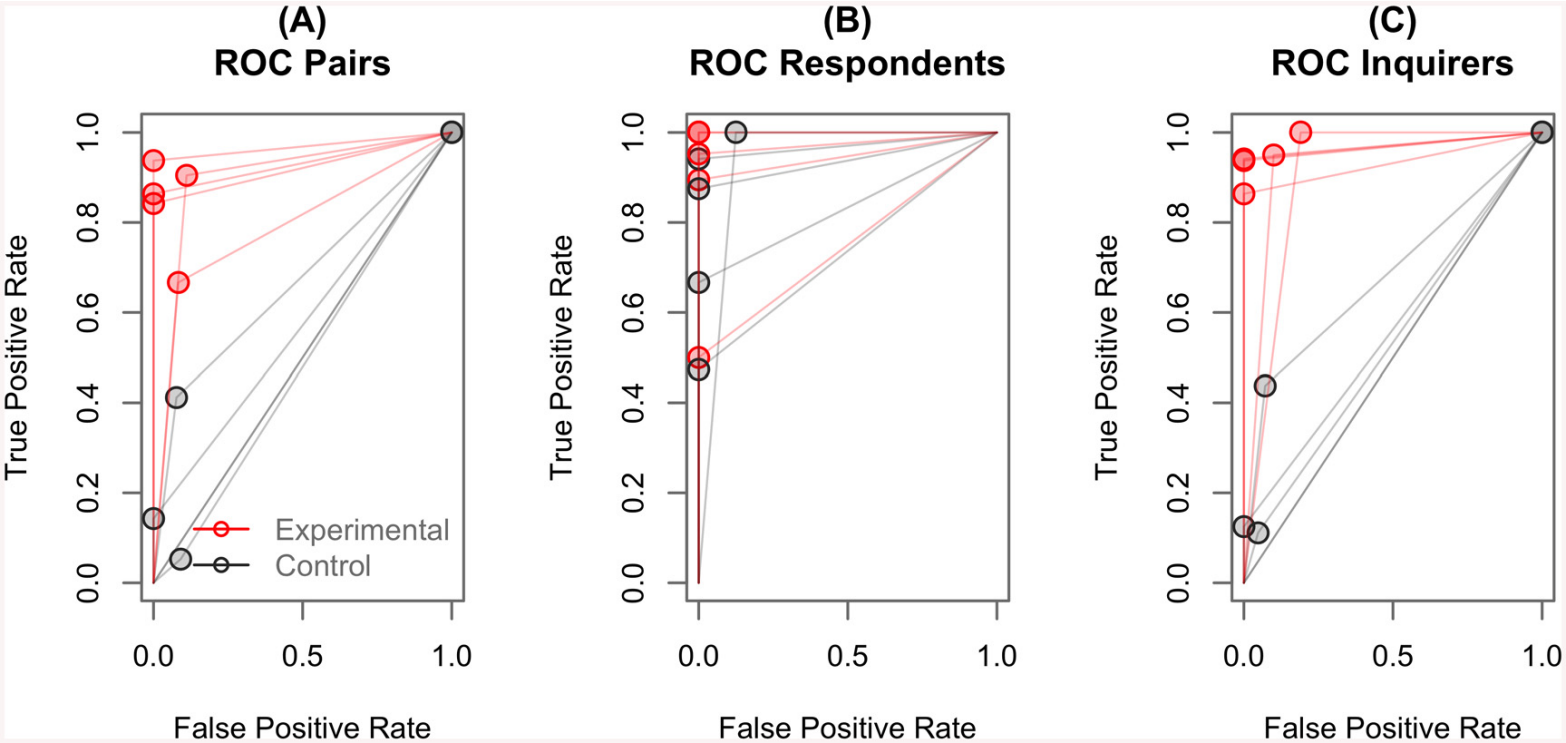
Performance of Each Pair of Subjects. Each plot illustrates the performance of pairs of subjects in terms of Receiver Operating Characteristic (ROC) for the experimental (red dots) and control (gray dots) games. The first plot (A) depicts the performance of each pair as whole while the other two plots represent the individual performances of respondents (B) and inquirers (C) in each pair. Overlapping points are indicated by darker shades of red or gray.

A more detailed way to examine our results is to analyze a BBI using signal detection theory, or equivalently, to treat the BBI as a classification problem. In this analysis, the signal (or, in classification terms, the label) is the true response to each question, and the prediction (or the class) is the inquirer’s understanding of the respondent’s answer. The performance of each pair can then be described using Receiver Operating Characteristic (ROC) analysis [18]. In ROC analysis, performance is represented as a point in the coordinate space defined by two axes representing the true positives rate and the false positives rate. In this space, an ideal classifier will occupy the upper-left corner of the plot, corresponding to 0% false positives and 100% true positives. A random classifier, by contrast, would occupy any of the positions along the upwards diagonal line, where for each proportion of true positives an equal amount of false positives occurs. For each pair in our experiments, we expect performance in the experimental condition to be near to the upper-left corner, and performance in the control condition to be near the diagonal line. Each pair’s performance can be further broken down into separate data points, one for the respondent (considering the true response as the ground truth and the output of the EEG detection as the prediction) and one for the inquirer (considering the TMS pulse as the ground truth and the inquirer’s decision as the prediction). Fig 5 plots each of these ROC points separately for each pair (Fig 5A) and for each participant within each pair (Fig 5B and 5C).

In the ROC plot, the performance associated with each point can be quantified as a single number, which represents the area of the polygon formed by connecting the point to the lower-left, lower-right, and upper-right corners of the plot, with coordinates (0, 0), (0, 1) and (1, 1), respectively [18]. This value is known as Area Under the Curve (AUC) and ranges from 1.0 (for an ideal classifier that recognizes all of the true positives without any false positives) to 0.5 (for a random classifier, where increasing the true positive rate produces an equivalent increase in the false positive rate [18]). Individual AUCs were calculated using ROCR package [19]. Since the distribution of AUC values was normally distributed (Shapiro-Wilk test, *W* > 0.84, *p* > 0.16) and had similar variance (Levene’s test, *F*(1, 8) = 0.05, *p* = 0.82) in both conditions, the data was analyzed with Student’s *T*-test. The mean raw AUC for the control condition was 0.54, a number that is not significantly different from that for chance (single-sample *t*(4) = 1.27, *p* = 0.27). On the other hand, the raw AUC for the experimental condition was 0.90, which is significantly larger than the AUCs for both the control condition (paired *t*(4) = 11.93, *p* = 0.0003, Cohen’s *d* = 8.44) and chance (single-sample *t*(4) = 13.43, *p* = 0.0001; see Fig 4B).

In the ROC plot, the performance associated with each point can be quantified as a single number, which represents the area of the polygon formed by connecting the point to the lower-left, lower-right, and upper-right corners of the plot, with coordinates (0, 0), (0, 1) and (1, 1), respectively [18]. This value is known as Area Under the Curve (AUC) and ranges from 1.0 (for an ideal classifier that recognizes all of the true positives without any false positives) to 0.5 (for a random classifier, where increasing the true positive rate produces an equivalent increase in the false positive rate [18]). Individual AUCs were calculated using ROCR package [19]. Since the distribution of AUC values was normally distributed (Shapiro-Wilk test, *W* > 0.84, *p* > 0.16) and had similar variance (Levene’s test, *F*(1, 8) = 0.05, *p* = 0.82) in both conditions, the data was analyzed with Student’s *T*-test. The mean raw AUC for the control condition was 0.54, a number that is not significantly different from that for chance (single-sample *t*(4) = 1.27, *p* = 0.27). On the other hand, the raw AUC for the experimental condition was 0.90, which is significantly larger than the AUCs for both the control condition (paired *t*(4) = 11.93, *p* = 0.0003, Cohen’s *d* = 8.44) and chance (single-sample *t*(4) = 13.43, *p* = 0.0001; see Fig 4B).

A final measure of interest is the number of bits that were successfully transferred between the respondent and the inquirer using the BBI. Because each answer of the respondent excludes half of the remaining options in the inquirer’s list, it theoretically conveys exactly one bit of information. Thus, information can be calculated as the sum of all the correct answers understood by the inquirer. This measure, however, might overestimate performance, as it does not account for the fact that negative answers are always correctly identified in the control conditions (that is, “0” bits are always transferred correctly), and that the distribution of the respondent’s answers might be skewed (i.e., there might be more negative answers than positive during the experimental conditions). A more conservative and precise measure is the Mutual Information (MI) between the ground-truth correct answers (considered as the input to the BBI) and the inquirer’s understanding of the received answers (the output of the BBI) [20]. MI can be calculated by first converting the set of correct answers and the inquirer’s responses into two binary vectors *C* and *R*, with every single answer *c* in *C* and *r* in *R* coded as 1 for “Yes” and “0” for “No”, and then using the following formula:

$$MI\left(C,R\right)\;=\;\sum_{c\in \left[0,1\right]}\sum_{r\in \left[0,1\right]}\text{log}\left(\frac{p\left(c,r\right)}{p\left(c\right)p\left(r\right)}\right)$$

MI was computed using the Infotheo package [21]. Because MI ranges between 0 and 1 and is calculated in natural logarithms, the actual number of bits transferred is calculated by first converting the MI value into the corresponding base two logarithm, and then multiplying it by the total number of answers given over the course of the experiment (in this case, 30). Because the distribution of MI values is inherently non-Normal (Shapiro-Wilk test, *W* = 0.78, *p* = 0.06), the raw data was square-root transformed before being analyzed. The transformed MI scores were more Normally distributed (Shapiro-Wilk test, *W* = 0.82, *p* = 0.12) and had comparable variances across conditions (Levene’s test, *F*(1,8) = 0.14, *p* = 0.71), thus fitting the requirements of Student’s *T*-test. In the experimental condition, the raw mean number of bits transferred was 16.02, which was significantly greater than the mean 1.16 bits transferred during the control condition (paired *t*(4) = 6.02, *p* = 0.004, Cohen’s *d* = 5.87 Fig 4C). The raw number of bits transferred in the experimental condition was also greater than chance, which, in the mutual information framework, corresponds to 0 bits (single-sample *t*(4) = 11.32, *p* = 0.0003). Contrastingly, the average of 1.16 bits transferred during the control condition was not significantly different than chance (single-sample *t*(4) = 1.88, *p* = 0.13).

In summary, the different measures of BBI performance collectively demonstrate that the inquirers correctly identified the respondents’ answers during the experimental conditions, but performed at chance levels during the control games. These results have two implications. First, the success of the five pairs during the experimental condition confirms that the interactive BBI was successful and generalizable across different human subjects. Second, the fact that the pairs’ performance was at chance levels during the control condition implies that the communication between the two participants was relying specifically on the information provided by the direct brain-to-brain connection, rather than through other environmental or sensory cues.

## Conclusions

This paper presents the successful demonstration of a new, non-invasive BBI in humans, which allowed pairs of participants to successfully collaborate and complete a series of question-and-answer games using information transferred between their brains. This BBI paradigm significantly extends and improves previous protocols in that (1) it involves the transfer of consciously perceived information in the form of phosphenes, (2) works in real-time, and (3) permits bidirectional information exchange between two participants. In this sense, this experiment represents a significant step forward towards the goal of creating BBIs with real-world applications. Because phosphenes are private to the receiver and can be perceived under a variety of conditions, or even while performing other actions, they represent a more versatile and interesting means of transferring information than the induced finger movements used in [5]. The amount of information transferred (as measured by the number of bits) in the current experiment is also significantly greater than that transferred in previous BBI experiments, e.g., close to 25 bits for one of the pairs (see Fig 4C) compared to a *maximum* of 13.4 bits in [5]. Finally, another strength of the current study is the enrollment of participants in terms of number and diversity. With ten total participants, this is the largest BBI experiment to date; while the number might seem small compared to more traditional EEG BCI studies, the reported effect sizes are considerable, with Cohen’s *d* values ranging from 5.87 to 9.55. As a comparison, these effect sizes would correspond to Pearson correlation coefficient values between 0.96 and 0.99. With such effect sizes, calculations suggest that between 3 and 4 pairs of subjects would already be sufficient to achieve a statistical power > 0.99 for significance thresholds of *p* < 0.05, therefore having less than 1% chance of incurring in Type II errors. Our sample size is therefore statistically adequate to support our conclusions. In addition, this is also the most diverse BBI experiment in terms of subject characteristics. For example, to our knowledge, this is the *first* human BBI experiment to include female participants. Thus, our results are encouraging in terms of their ability to demonstrate the generalizability of BBIs to the general population.

The current experiment is not without limitations. The most obvious of these is that, as in previous BBI demonstrations, our paradigm does not transfer information better than would be expected with canonical means, such as when two individuals communicate verbally. For instance, it is reasonable to assume that any pair of our participants would be able to correctly guess the object in 100% of the games if they were given the possibility to communicate verbally. However, in circumstances including a non-verbal participant (e.g., an individual with Broca’s aphasia), or pairs of participants that do not speak the same language, it is highly likely that the BBI paradigm would outperform a canonical verbal protocol.

Note that the count of correct final guesses greatly underestimates the performances of individual participants. This is for two reasons; first, all the answers in a game need to be correct for a correct guess of the chosen object; and second, the accuracy of a pair is the product of the accuracies of its two independent participants. When the accuracies of each respondent and inquirer in identifying each response are considered, the results are indeed impressive (93.5% and 94.0% accuracies, respectively), with two participants achieving a perfect score (Respondents 2 and 3, in the Experimental condition). These performances correspond to a decline of only about 6% from the levels assessed at the end of the training phase for the inquirers. This small decline can be explained by mental fatigue, which was likely induced by the length of the experimental sessions (approximately 2 hours for the respondents, and 2.5 hours for the inquirers) and the need for participants to fixate the screen for long periods of time. There is, in fact, experimental evidence suggesting that reduced attention has detrimental effects on both SSVEP detection [22] and phosphene perception [23].

A second limitation of our study is that, while the experimental paradigm as a whole allowed bi-directional transfer of information between the respondent and the inquirer, the BBI remained *unidirectional*, allowing only the transfer of information from the respondent to the inquirer. Fully bi-directional BBIs, if executed in real-time, require the integration of EEG and TMS technologies for each participant, and the development of tasks in which all of the relevant information can be transmitted solely using the BBI. A final limitation is that the code used herein to transmit information was relatively simple, essentially relying on the transmission of binary perceptual signals.

None of these limitations is inherent to the technologies or paradigms we adopted for the BBI. For example, EEG and TMS [24], or alternately fMRI and TMS [25], can be used concurrently, thus enabling true bidirectional BBIs. We intend to investigate such BBIs in the future. Our current efforts are aimed at increasing the complexity of information transmitted through human BBIs, such as the transmission of affective states and other types of information that would be otherwise difficult to verbalize. Under these circumstances, larger amounts of information could potentially be transferred, and a BBI would confer a distinct advantage over canonical, symbolic means of communication. We see this as an exciting venue for future research.
